# Diversification of MIF immune regulators in aphids: link with agonistic and antagonistic interactions

**DOI:** 10.1186/1471-2164-15-762

**Published:** 2014-09-05

**Authors:** Géraldine Dubreuil, Emeline Deleury, Didier Crochard, Jean-Christophe Simon, Christine Coustau

**Affiliations:** Sophia Agrobiotech Institute, INRA-CNRS-UNS, UMR 7254, 400 Route des Chappes, 06 903 Sophia Antipolis, France; Institut de Recherche sur la Biologie de l’Insecte, UMR 7261, CNRS/Université François-Rabelais, Parc Grandmont, 37200 Tours, France; Institute of Genetics, Environment and Plant Protection, INRA, UMR 1349, Domaine de la Motte, BP 35327, 35653 Le Rheu Cedex, France

**Keywords:** Invertebrate immunity, Cytokines, Macrophage migration inhibitory factor, Host-parasite interactions, Host-symbiont interactions, *Acyrthosiphon pisum*

## Abstract

**Background:**

The widespread use of genome sequencing provided evidences for the high degree of conservation in innate immunity signalling pathways across animal phyla. However, the functioning and evolutionary history of immune-related genes remains unknown for most invertebrate species. A striking observation coming from the analysis of the pea aphid *Acyrthosiphon pisum* genome is the absence of important conserved genes known to be involved in the antimicrobial responses of other insects. This reduction in antibacterial immune defences is thought to be related to their long-term association with beneficial symbiotic bacteria and to facilitate symbiont maintenance. An additional possibility to avoid elimination of mutualistic symbionts is a fine-tuning of the host immune response. To explore this hypothesis we investigated the existence and potential involvement of immune regulators in aphid agonistic and antagonistic interactions.

**Results:**

In contrast to the limited antibacterial arsenal, we showed that the pea aphid *Acyrthosiphon pisum* expresses 5 members of Macrophage Migration Inhibitory Factors (ApMIF), known to be key regulators of the innate immune response. *In silico* searches for MIF members in insect genomes followed by phylogenetic reconstruction suggest that evolution of MIF genes in hemipteran species has been shaped both by differential losses and serial duplications, raising the question of the functional importance of these genes in aphid immune responses. Expression analyses of ApMIFs revealed reduced expression levels in the presence, or during the establishment of secondary symbionts. By contrast, *ApMIFs* expression levels significantly increased upon challenge with a parasitoid or a Gram-negative bacteria. This increased expression in the presence of a pathogen/parasitoid was reduced or missing, in the presence of facultative symbiotic bacteria.

**Conclusions:**

This work provides evidence that while aphid’s antibacterial arsenal is reduced, other immune genes widely absent from insect genomes are present, diversified and differentially regulated during antagonistic or agonistic interactions.

**Electronic supplementary material:**

The online version of this article (doi:10.1186/1471-2164-15-762) contains supplementary material, which is available to authorized users.

## Background

Invertebrate immune systems have been shown to include highly diversified recognition systems, complex regulatory processes, and specific effectors [1–5]. However, the functioning and evolutionary history of immune-related genes remains unknown for most invertebrate species.

For example, the recent sequencing of the first aphid genome (*Acyrthosiphon pisum*) provided new insights into insect immunity but also raised novel evolutionarily- and functionally-intriguing questions [6]. Indeed, genes participating in the immune responses of other insects are missing in aphids, such as the microbial recognition genes PGRPs (peptidoglycan receptor proteins) involved in the recognition of both Gram-negative and -positive bacteria [7, 8]. Even more intriguing is the absence of most of the components of the immunodeficiency (IMD) signaling pathway, which is critical for fighting Gram-negative bacteria, and the absence of most of the antimicrobial peptides conserved in other insects [7, 8]. These results, further supported by expression studies [8], showed that the aphid immune system greatly differs from that of other well studied insects and it was hypothesized that aphids compensate for a deficient immune system by symbiont-mediated host protection and an extraordinary reproduction rate [8]. Aphids are plant sap-feeding insects associated with the obligatory nutrient-providing symbiont *Buchnera aphidicola* [9, 10]. Aphids also interact with several species of facultative bacterial endosymbionts [11] that are found free in the hemolymph [11, 12]. Interestingly, secondary symbionts have been shown to influence important fitness-related traits such as body pigmentation [13], offspring production [14] and resistance to parasitoids [15–17] or pathogens [18]. It has been hypothesized that these intimate aphid-symbiont interactions may have led to the loss of specific antibacterial compounds or pathways to accommodate long-term host-symbiont coevolution and symbiont maintenance [19].

An additional possibility to avoid elimination of mutualistic symbionts is a fine-tuning of the host immune response [20, 21]. Aphid immune system may be particularly well regulated to prevent or limit damage to their bacterial mutualists under pathogen infections. As a first approach to explore this hypothesis, we searched *A. pisum* genome for immune regulators, and we noticed the existence of five genes coding for Macrophage Migration Inhibitory factors (MIFs) within the *A. pisum* genome. In vertebrates, MIFs are important pro-inflammatory cytokines acting on key cellular processes of the immune response such as cell proliferation and apoptosis [22, 23]. MIFs were identified in a variety of species, including protozoan, nematode, mollusk and crustacean species [5, 24–29]. It was shown that a mollusk MIF (BgMIF1) not only presented the expected activities on cell proliferation and apoptosis but played a major role in the response against parasitic infection [5]. This study provided functional evidence of the conservation of major immune-related functions of MIFs in an invertebrate and highlighted the importance of these immune regulators in invertebrate immunity [5]. Interestingly, all species investigated so far presented one or two MIF gene copies. The existence of additional MIF copies in *A. pisum* therefore requires dedicated evolutionary and functional analyses to better understand the complex immune interactions of aphids with their symbionts and pathogens.

Here we characterized *A. pisum* MIF members and explored their existence and evolutionary history in various insect phyla. To gain insights into their functional role, we analyzed the expression of the several MIF copies during aphid-symbiont-pathogen interactions.

## Results

### *Acyrthosiphon pisum*MIF (*ApMIF)*family members

The search for predicted *MIF* gene sequences in the annotated genome of *Acyrthosiphon pisum* [6] (Acyr_2.0 assembly) revealed 5 MIF members referred to as ApMIF1 to ApMIF5 (Table 1). Using BLAST searches [30], we found one or several full-length ESTs for each ApMIF member supporting their existence, structural annotation and expression. Complementary searches evidenced an additional hit, corresponding to a 6th *MIF* gene that was actually annotated in a previous version of the genome assembly (Acyr_1.0) (Table 1). BLAST searches using the originally predicted ApMIF6 cDNA sequence against the 214920 redundant ESTs resulted in a partial and fragmented alignment with only 4 similar ESTs that did not support the existence of a full length transcript from a 6th MIF gene. In order to further characterize ApMIF members, RT-PCR amplification of the complete coding sequences of the predicted ApMIF genes was performed from RNA of two genetically different lineages of *A. pisum* (LL01 and YR2). Amplicons were obtained and resequenced for ApMIF1 to -5. Sequence results confirmed the expression of full length transcripts of ApMIF1 to ApMIF5. Amplification of a full length cDNA from ApMIF6 (based on originally predicted sequence) was however not obtained.

**Table 1:** **Accession numbers and summary information on ApMIF genes and their products**

	Aphidbase	NCBI	NCBI	NCBI	PF01187	Exp. validation
	Genes	Genes	RefSeq mRNA	RefSeq proteins	e-value	
ApMIF1	ACYPI002465	LOC100161225	NM_001162635.1	NP_001156107.1	1.53e-38	Yes
ApMIF2	ACYPI006088	LOC100165124	XM_001946905.2	XP_001946940.1	5.85e-24	Yes
ApMIF3	ACYPI000036	LOC100144890	NM_001126157.2	NP_001119629.1	4.61e-25	Yes
ApMIF4	ACYPI003547	LOC100162394	NM_001162060.1	NP_001155532.1	2.51e-24	Yes
ApMIF5	ACYPI005907	LOC100164929	XM_001948047.2	XP_001948082.1	6.43e-15	Yes
ApMIF6	ACYPI002954	LOC100161756	Withdrawn	Withdrawn	-	No

Experimental validation (Exp. validation) consisted in the amplification and resequencing of cDNAs.

The five predicted ApMIF proteins showed highly significant hits with Macrophage Migration Inhibitory factor domain (PF01187/IPR001398) (Table 1). Interestingly, sequences of the 5 ApMIF predicted proteins differ considerably among each other (Additional file 1: Figure S1) and present 33% to 55% similarity in their sequences. MIF proteins from mammals and snails have been shown to catalyze the ketoenol isomerization of small aromatic substrates such as hydroxyphenylpyruvate and L-dopachrome methyl ester [5, 31]. Post-translational cleavage of the initiating methionine exposes an N-terminal catalytic proline (Pro2) that is essential for MIF tautomerase activity [32]. Three of the five predicted ApMIF proteins present this Proline2 residue as well as other conserved residues such as Lys33, Ile65 and Tyr93 and Val107, participating to MIF active sites [33] (see Additional file 1: Figure S1 for alignment of ApMIF sequences). Another conserved feature of MIF proteins is to be secreted via non-classical pathways, involving, for example an ATP binding cassette transporter [34]. As expected, no signal peptides were detected in ApMIF sequences (SignalP 4.0; [35]) but the *SecretomeP* server [36] predicted ApMIF 1 and ApMIF 2 as secreted proteins (secP score 0.934 and 0.78 respectively).

### MIF family members in insects

In order to further characterize this multigenic family, we first searched for *MIF* genes in sequenced insect genomes. Intriguingly, with the exception of two *MIF* genes in *Tribolium castaneum* and in *Bombyx mori*, no *MIF* genes were identified from the other insect genomes available at the time of the study (Table 2). We then performed an extensive search for MIF transcripts from 27 hemipteran species (Table 3) with available ESTs in public databases. Interestingly, MIF transcripts were detected, in particular, in phloem-sap feeding species belonging to the Delphacidae, Aphididae, Pemphigidae, Pseudococcidae and Psyllidae families (Table 3). MIF genes were identified in all Aphididae with available ESTs with up to 5 members. Note that except for *A. pisum* the number of MIF members is probably underestimated due to the absence of annotated genomic data and to the modest size of some of the transcriptomic databases. Aphid genomic and transcriptomic data are currently growing rapidly and will allow a complete identification of aphid MIF members in a close future.

**Table 2:** **Survey of the number of MIF genes in insect genomes**

	Species	Nb MIF
*Diptera*	*Drosophila species* (r5.46, ftp://ftp.flybase.org)	0
	*Anopheles gambiae* (r3.6, ftp://ftp.flybase.org)	0
	*Aedes aegypti* (r1.3, http://aaegypti.vectorbase.org/)	0
*Lepidoptera*	*Bombyx mori* (http://www.silkdb.org/)	2
*Coleoptera*	*Tribolium castaneum* (v2.0, http://www.hgsc.bcm.tmc.edu/)	2
*Hymenoptera*	*Acromyrmex echinatior* (v3.8, http://www.antgenomes.org/)	0
	*Apis melifera* (r4.5, http://hymenopteragenome.org/beebase/)	0
	*Apis florea* (v1.0, http://www.hgsc.bcm.tmc.edu/)	0
	*Bombus impatiens (v2.0,* http://hymenopteragenome.org/beebase/?q=gbrowse_bimp *)*	0
	*Bombus terrestris (v1.1,* http://www.hgsc.bcm.tmc.edu/ *)*	0
	*Nasonia vitripennis (v0.5,* http://www.hgsc.bcm.tmc.edu/ *)*	0
*Hemiptera*	*Acyrthosiphon pisum (v2.1,* http://www.aphidbase.com *)*	5
*Anoplura*	*Pediculus humanus (u1.2,* https://www.vectorbase.org/ *)*	0
*Cladocera*	*Daphnia pulex (v1.0,* http://genome.jgi.doe.gov/pages/blast.jsf?db=Dappu1 *)*	3

Between parentheses are indicated the assembly version numbers and the URL used for genomic database searches. *Daphnia pulex* is added as an outgroup.

**Table 3:** **Survey of complete MIF family members from 27 available hemiptera species**

***Order***	***Suborder***	***Family***	***Species***	***Num EST***	***Nb MIF***
**Hemiptera**	Auchenorryncha	Cicadellidae	*Graphocephala atropunctata*	6481	0
			*Homalodisca vitripennis*	20030	0
			*Oncometopia nigricans*	9056	0
	Heteroptera	Delphacidae	*Nilaparvata lugens (*Nl)	118020	2
			*Peregrinus maidis (*Pm)	20678	2
		Cimicidae	*Cimex lectularius*	7129	0
		Miridae	*Adelphocoris lineolatus*	2915	0
		Reduviidae	*Dipetalogaster maximus*	2671	0
			*Rhodnius prolixus*	16105	0
			*Triatoma brasiliensis*	2115	0
			*Triatoma infestans*	2564	0
			*Triatoma matogrossensis*	2230	0
			*Triatoma rubida*	1850	0
		Lygaeidae	*Oncopeltus fasciatus*	1115	0
	Sternorrhyncha	Aleyrodidae	*Bemisia tabaci*	11923	0
		Aphididae	*Aphis gossypii* (Ag)	88851	2
			*Rhopalosiphum maidis* (Rm)	17649	4
			*Rhopalosiphum padi* (Rp)	17892	3
			*Toxoptera citricida* (Tc)	4304	2
			*Acyrthosiphon kondoi* (Ak)	23040	2
			*Acyrthosiphon pisum* (Ap)	214834	5
			*Myzus ascalonicus* (Ma)	22942	5
			*Myzus persicae* (Mp)	27728	3
			*Sitobion avenae* (Sa)	3478	1
		Pemphigidae	*Pemphigus spyrothecae*	18587	0
		Pseudococcidae	*Maconellicoccus hirsutus* (Mh)	7669	2
		Psyllidae	*Diaphorina citri* (Dc)	19598	1

The total number of redundant ESTs (Num EST) available in public databases at the time of the study is shown for each species, as well as the number of complete cDNA unique sequences predicted to code MIF proteins (presence of PF01187 domain confirmed by pfam_scan). Between parentheses are shown the species identification letters as used for the phylogenetic reconstruction.

### Phylogenic reconstruction of MIFs from hemipterans

**Figure 1:**
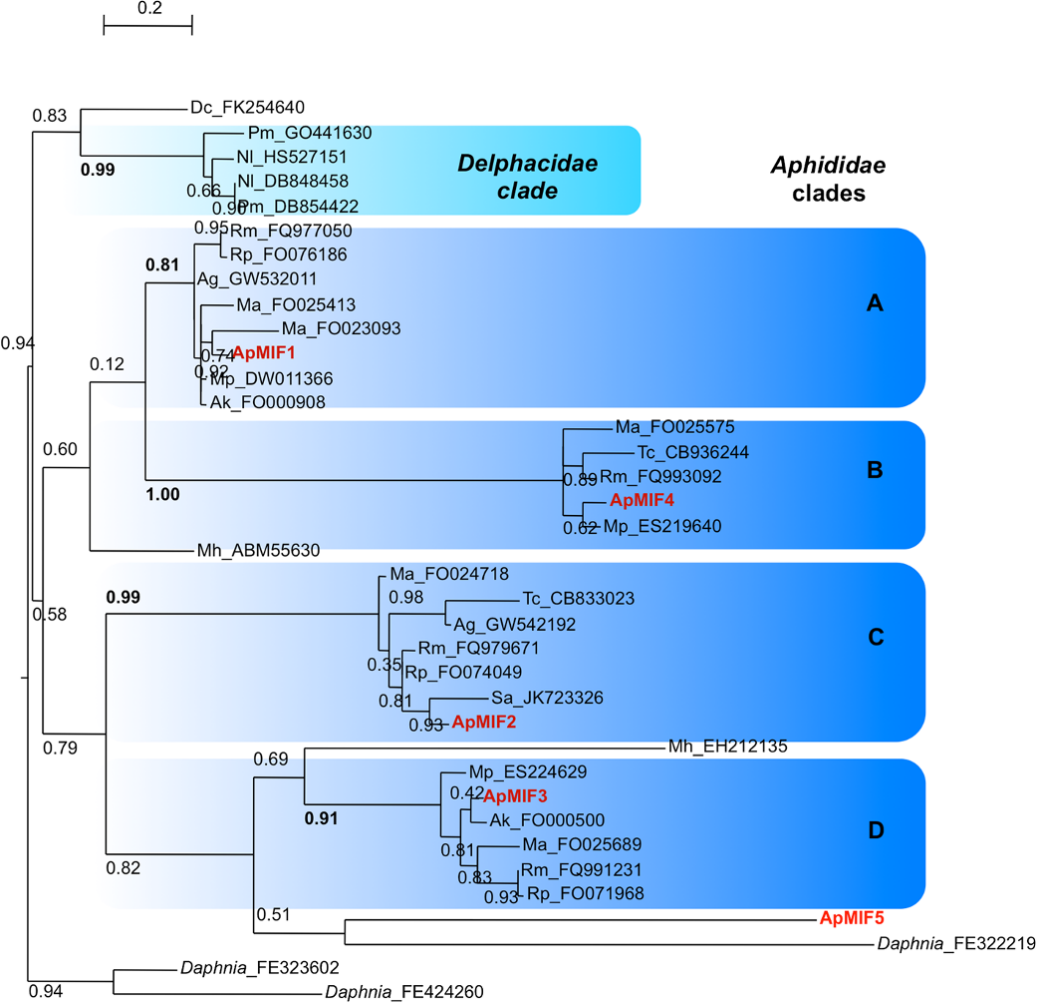
**Maximum likelyhood tree showing the phylogenetic relationship between full-length hemiptera MIF unisequences (listed in Table** 3 **).** Genbank accession numbers of the sequences used for the reconstruction are shown next to the species identification letters (2 letters as defined in Table 3). Clades that are specific of delphacidae or aphididae **(A-D)** are highlighted in light and dark blue, respectively. Values at nodes are bootstrap proportions. Bar: 0,2 substitution/site.

Phylogenic reconstruction of complete predicted MIF sequences from hemipterans was performed using the crustacean *Daphnia pulex* as an outgroup (Figure 1). With the exception of *A. pisum* ApMIF5, all predicted MIF proteins from Delphacidae and Aphididae clustered into five clades including one clade specific of Delphacidae and 4 clades specific of Aphididae (Figure 1). Each ApMIF member clustered into one of the four clades, together with MIF members from other aphid species (Figure 1), showing that several duplications occurred before the diversification of aphids but after the split of Aphididae from the other hemipterans.

### *ApMIFs*constitutive expression

**Figure 2:**
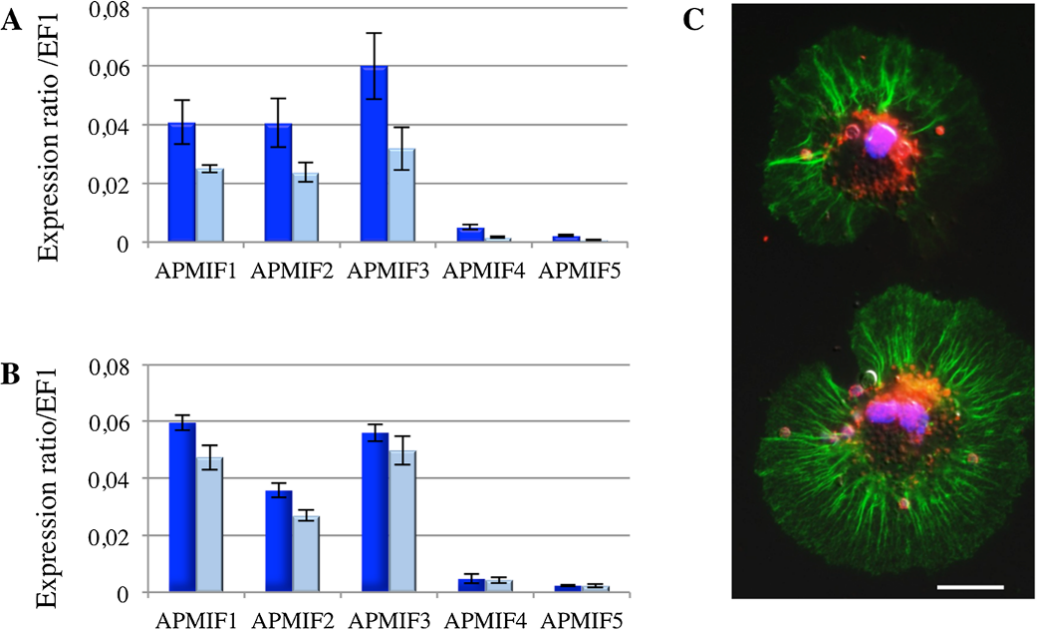
**Expression ratios (normalized to EF1 expression) of the five** ***ApMIF*** **transcripts in whole bodies (A) and hemocytes (B) from the** ***A. pisum*** **genetic lineages LL01 (dark blue) and YR2 (light blue).** Each bar represents the mean expression ratio ± SD obtained from three independent experiments. **(C)** Immunolocalization of ApMIF1 in hemocytes. Merger of fluorescent micrographs showing two hemocytes with labeled actin (green fluorescence), nuclei (purple/bleu fluorescence) and ApMIF1 protein (orange fluorescence). Scale bar represents 10 μm.

Expression of the 5 *ApMIF* transcripts was analysed by real time RT-PCR on 12-day-old aphids from YR2 and LL01 free of any secondary symbionts [14, 37]. In both lineages, transcripts of *ApMIF1*, *ApMIF2* and *ApMIF3*, respectively clustered in clade A, C and D, were highly detected in whole bodies while *ApMIF4* and *ApMIF5* showed very weak expression levels (Figure 2). All five *ApMIFs* transcripts were detected in hemocytes, supporting their potential involvement in immune processes (Figure 2B). To better assess the presence of ApMIFs in hemocytes, we used an antibody raised against peptides of one of the well-expressed ApMIFs (ApMIF1). As shown in the Figure 2C, ApMIF1 protein localizes within hemocyte granules.

Further studies on ApMIF expression were carried out on the 5 ApMIFs. However, because expression levels of *ApMIF4* and *ApMIF5* remained at the limit of detection by real time quantitative RT-PCR in each experiment, they are not presented in the following result sections.

### Increased *ApMIF*expression after immune challenges

**Figure 3:**
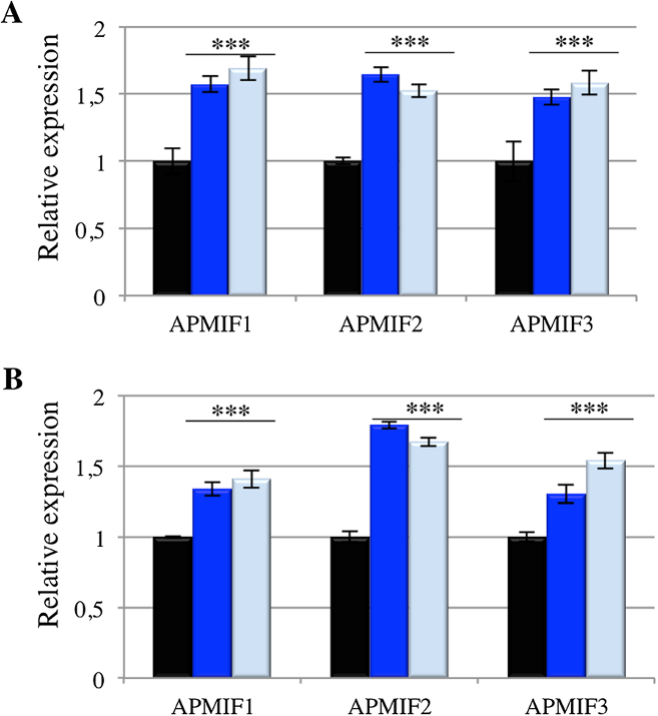
**Relative expression (normalized to EF1 expression and to expression in control aphids (black)) of the three well-expressed** ***ApMIF*** **s transcripts, three days after immune challenge by** ***Aphidius ervi*** **(dark blue) or** ***E. coli*** **(light blue) in the** ***A. pisum*** **lineages LL01 (A) and YR2 (B).** Each bar represents the mean expression ± SD obtained from three independent experiments. Significant differences as compared with control are indicated by stars (Anova on row data: ***p < 0.0002).

To explore the expected involvement of *ApMIF* genes in aphid immune response, we examined their expression levels after immune challenges. We first used *Aphidius ervi,* which is the main parasitoid of *A. pisum* in the field [38]. The two clones of *A. pisum* showed a significant increase in *ApMIF1*, *ApMIF2* and *ApMIF3* transcript levels after exposure to female parasitoid (Figure 3). An increased *ApMIF* expression is also observed in aphids after injection with the Gram-negative bacteria *E. coli* (Figure 3).

### Decreased *ApMIF*expression during interactions with mutualistic symbionts

**Figure 4:**
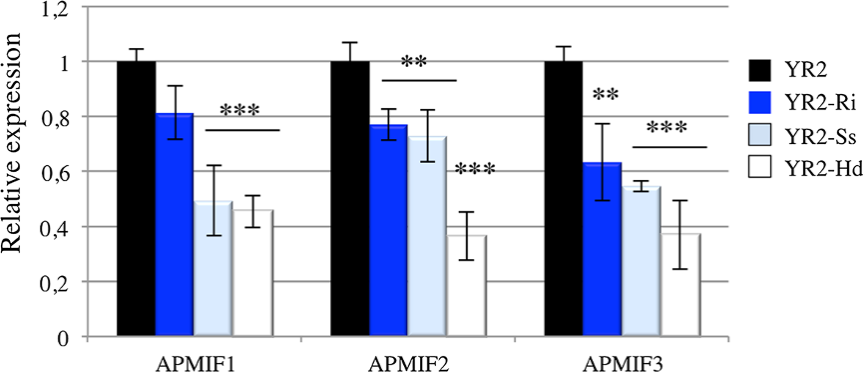
**Comparison of** ***ApMIFs*** **expression in YR2 aphids associated with** ***R. insecticola*** **(YR2-Ri),** ***S. symbiotica*** **(YR2-Ss) or** ***H. defensa*** **(YR2-Hd).** Expression has been normalized to EF1 expression and to expression in control aphids (black). Each bar represents the mean expression ± SD obtained from three independent experiments. Significant differences as compared with control are indicated by stars (Anova on row data: ***: p < 0.0002; **: p < 0.002).

**Figure 5:**
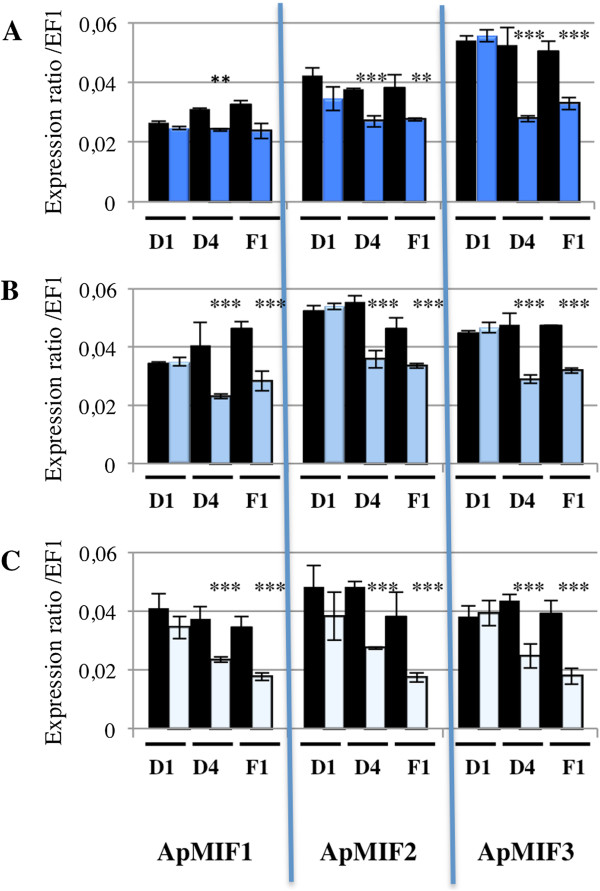
**Effect of secondary symbiont injection on** ***ApMIFs*** **expression in LL01 aphids injected with** ***R. insecticola*** **(A),** ***S. symbiotica*** **(B) or** ***H. defensa*** **(C).** Expression was measured at one (D1) or four (D4) days post injection, as well as in offspring from the first generation (F1), in symbiont injected (blue) and buffer injected (black) aphids. Significant differences as compared with control are indicated by stars (Anova on row data: ***: p < 0.0002; **: p < 0.002).

We first compared the expression levels of *ApMIF* transcripts in the three lineages derived from YR2 and each harbouring a specific secondary symbiont (YR2-Hd, YR2-Ri and YR2-Ss). Aphids carrying mutualistic symbionts showed lower expression levels of *ApMIF1*, *ApMIF2* and *ApMIF3* (Figure 4). In order to further study changes in *ApMIF* expression in presence of secondary symbionts, we measured *ApMIF* expression levels in LL01 following the injection of each of the three secondary symbionts. Since it was reported that injection of hemolymph from infected to uninfected *A. pisum* can establish a stable infection of secondary facultative symbionts [39, 40], each secondary symbiont was manually collected from YR2 lineages and injected into LL01 adults. *ApMIF* expression was measured 1 and 4 days after injection. *ApMIF1*, *ApMIF2* and *ApMIF3* expression levels rapidly decreased after infection as compared to controls injected with Schneider medium (Figure 5). To ascertain that experimental infections with the facultative symbionts were successful, individuals from the F1 progeny of injected adults were microscopically examined. Presence of bacteria in the hemolymph confirmed the establishment of these vertically transmitted symbionts, and analyses of *ApMIFs* expression levels confirmed that their decreased expression persisted in the F1 progeny (Figure 5). Altogether, these results showed that the establishment and the presence of the three major facultative symbionts of *A. pisum* correlate with a decreased expression of the three well-expressed *ApMIF* genes.

### *ApMIF*expression during both agonistic and antagonistic interactions

**Figure 6:**
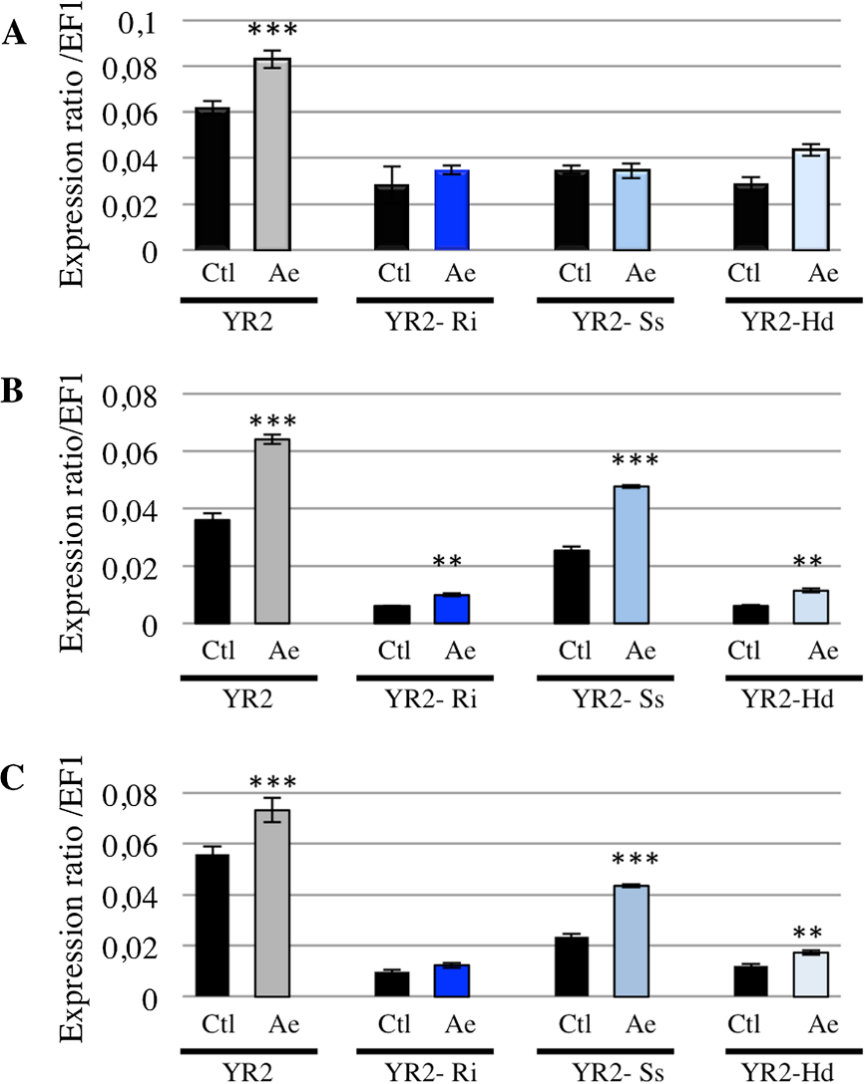
**Expression levels of** ***ApMIF1*** **(A),** ***ApMIF2*** **(B) and** ***ApMIF3*** **(C) in the YR2 lineage without symbiont (YR2) or associated with** ***R. insecticola*** **(YR2-Ri),** ***S. symbiotica*** **(YR2-Ss) or** ***H. defensa*** **(YR2-Hd), at 3 days post-exposure to the parasitoid** ***A. ervi*** **(Ae).** Significant differences in expression as compared with control aphids (ctl) not exposed to *A. ervi* are indicated by stars (Anova on row data: ***: p < 0.0002; **: p < 0.002).

Because some secondary symbionts can impact important aphid fitness-related traits such as resistance to parasitoids [15–17] or pathogens [18], it may be disadvantageous for aphids to mount a complete immune response in the presence of both these mutualists and a pathogen or parasitoid. Similarly, if the negative regulation of aphid immune response results from a direct effect of mutualists on host immune system, one may expect that this regulation would be maintained after an immune challenge and would limit the increase in *ApMIF* expression. To test this hypothesis, *ApMIF* expression levels were analyzed after exposure to parasitoids in the YR2 lineages carrying each of the three facultative symbionts. No significant change in gene expression was observed for *ApMIF1* (Figure 6A). Increased expressions were observed for ApMIF2 and ApMIF3 after exposure to the parasitoid *A. ervi* (Figure 6B,C), the highest expression levels being observed in the YR2-Ss lineage. However, all expression levels remained far below those of the YR2 lineage without mutualistic symbionts (Figure 6).

## Discussion

Widespread lineage-specific gene duplications have been observed in the *A. pisum* genome [6]. The number of duplications in this aphid appears greater than that of any other sequenced insect with over 2000 gene families that have undergone gene duplication [6]. However, these duplications were not reported to particularly affect immune-related genes, and, on the contrary, important and conserved genes of the antibacterial pathways are missing from *A. pisum* genome [6].

A specific search for MIF genes in genomes from insects revealed that except for *Tribolium castaneum* and *Bombyx mori*, no other genome contained MIF members. Conversely, analysis of hemipteran transcriptomic databases provided evidence for the existence of a MIF multigenic family in Aphididae species as well as for the existence of at least 2 MIF members in Delphacidae species. Phylogenic reconstruction of complete predicted MIF sequences from hemipterans showed that sequences clustered into five clades including one clade specific of Delphacidae and 4 clades specific of Aphididae*.* Each Aphididae clade contained closely related MIF members from several aphid species showing that duplications occurred before the diversification of aphids. Altogether, these results showed that losses occurred in most insect phyla while both differential losses and duplications occurred within the hemiptera phylum. This differential loss versus duplication, together with the previously observed absence of important immune-related genes in *A. pisum* genome, raised the question of the functional significance of MIF members in Aphididae.

Detection of the ApMIF transcripts in aphid whole bodies and hemocytes is consistent with the expected expression of these cytokines. Mammalian MIFs are expressed in various cell types, including all immune-relevant tissues (e.g. lung and digestive epithelia, skin) and macrophages [22]. In mammals, MIF genes are constitutively expressed and the protein is stored in granules of immune-relevant cells and rapidly secreted in extracellular fluids upon challenge [41, 42]. Here we showed that ApMIF proteins are also detected in immune cell granules, suggesting that they are stored and secreted upon challenge. In addition to the expression localization, the significant increase in ApMIF expression after immune challenges support the role of these genes in promoting and regulating major early processes of the innate immune response [22]. MIFs are pleiotropic proteins involved in numerous cellular processes and are key cytokines controlling the response to both parasitic and bacterial infections [5, 22, 43, 44]. The over-expression of ApMIFs was observed in two distinct aphid clones and after exposure to both parasitoids and pathogenic bacteria, supporting a general involvement of ApMIFs in aphid’s immune response, regardless of the aphid genetic background or of the pathogen.

Interestingly, a lower expression of *ApMIF* genes was observed either constitutively or after immune challenge in aphid lineages infected by one of the three tested secondary symbionts (*R. insecticola*, *S. symbiotica* and *H. defensa)*. A recent study showed that the number of *A. pisum* hemocytes is significantly smaller in YR2 lineages carrying these secondary symbionts as compared with the YR2 lineage without secondary symbionts [12]. The decreased expression levels of MIF genes that we observed in these YR2 lineages may therefore result partly from their smaller hemocyte number and partly from a down regulation of expression.

These results are consistent with previous observations on the association between the weevil *Sitophilus* and the *Sitophilus* primary endosymbiont (SPE) [45]. In contrast to aphids, weevil immune system presents several families of conserved antimicrobial peptides [45] and it was shown that the antimicrobial peptide coleoptericin-A (ColA) inhibits bacterial cell division and is essential in the regulation of endosymbiont number and location [19]. In addition, although expression of antimicrobial peptides (AMP) was similar in symbiotic (carrying SPE) and aposymbiotic larvae, a lower AMP gene expression was observed after immune challenge with *E. coli* in symbiotic insects [45]. Here, we observed a negative effect of symbionts on expression of another type of immune genes in aphids, further supporting the importance of immune gene expression in regulating both agonistic and antagonistic interactions.

## Conclusions

The present study shows that members of the Macrophage Migration Inhibitory Factor (MIF) present a complex evolutionary history characterized by differential losses and duplications across insect phyla.

The 5 MIF members of *A. pisum* are functional, expressed in circulating immune cells and differently regulated during a pathogenic or mutualistic interaction.

This work provides evidence that while aphid’s antibacterial arsenal is reduced, immune regulators widely absent from insect genomes are present, diversified and differentially regulated during antagonistic or agonistic interactions. This supports the hypothesis of a fine-tuning of the immune response possibly accommodating both symbiont maintenance and response to aggressors.

## Methods

### Database searches and sequence analyses

Protein sequences encoded by MIF genes in *A. pisum* genome were identified using BLAST programs [30] at the Aphidbase (available at http://www.aphidbase.com/) and National Centre for Biotechnology Information (NCBI) servers, providing a 6.2X-coverage genome assembly (Acyr_2.0 and Acyr_1.0) of *A. pisum* [6]. Other public databases used included the Reference Sequence (RefSeq) database [46] for *A. pisum* mRNA and protein sequences and the 214,920 *A. pisum* ESTs available at the time of the study in NCBI-dbEST.

An exhaustive search of Hemiptera MIF transcripts in the NCBI’s databases (NR, dbEST) was performed using the BLAST program [30]. The predicted translation of each full-length or partial sequence was checked for the presence of the conserved MIF domain (PFAM reference number PF01187 and/or InterPro reference number IPR001398) using pfam_scan software (available at http://pfam.sanger.ac.uk/search). Quality of sequences and their annotations was validated by multiple alignments using Muscle [47]. Only full-length proteins were then used, re-aligned with Muscle and cleaned using Gblocks (version 0.91b, available at http://www.phylogeny.fr). A maximum likelihood phylogenetic tree was generated using PHYML sofware (version 3) [48] and the WAG model of amino acids subsititution matrice. Bootstrap values were obtained from 1000 replicate samples using the proportion of invariable sites and gamma distribution parameters defined by PHYML.

### Aphid lineages

Two distinct clones of *A. pisum* were used in this study, LL01 [37] and YR2 [14], both carrying only the obligate symbiont *Buchnera aphidicola*. For YR2, we also used 3 different lineages derived from the same genetic background but each differing by their respective secondary symbionts, being *Regiella insecticola* (U-type or PAUSS), *Hamiltonella defensa* (T-type or PABS), or *Serratia symbiotica* (R-type or PASS) [14]. These lineages are referred to as YR2-Ri, YR2-Hd and YR2-Ss, respectively.

Aphids were reared on *Vicia faba* under long photoperiod (16 hours light/8 hours dark) and temperature (19°C) conditions to maintain continuous parthenogenetic reproduction [49]. Parthenogenetic wingless adults (12–15 days old) were used for all experiments except for parasitism by *Aphidius ervi* that was carried on 3 days-old larvae.

### Hemocyte collection

Hemolymph was collected in a total volume of 200 μL of aphid Schneider medium (Sigma-Aldrich), as previously described [12]. Hemolymph was centrifuged at 200 g for 5 minutes to eliminate putative embryos and hemocyte-containing supernatant was either used for total RNA extraction or further processed for immunolabeling.

### Immunolabeling of ApMIF1 in *A.pisum*hemocytes

Hemocytes were fixed in paraformaldehyde 4% in phosphate buffer 0.1 M pH7.2 for 10 minutes and washed 3 times in PBS under agitation. Cells were then permeabilized with Triton X100 at 0.1% in PBS for 10 minutes and washed 3 times with PBS under agitation. Hemocytes were incubated with a custom-made antibody raised against two ApMIF1 peptides (shown in the Additional file 1: Figure S1) (diluted 1/1000 in PBS1X-0.05% tritonX100) for 1 hour at room temperature. Control hemocytes were incubated with rabbit pre-immune serum (diluted 1/1000 in PBS1X-0.05% Triton X100). After three washes in PBS, hemocytes were incubated for 1 hour at room temperature in anti-rabbit Alexa Fluor 488 secondary antibody (diluted 1/500 in PBS1X-0.05% Triton X100) and anti-phalloidin-X5-FluoProbe (diluted 1/200 in PBS-0.05% Triton X100) to detect actin cytoskeleton. After 3 washes in PBS, nucleuses were marked by incubation in DAPI (1 μg/mL) for 10 minutes. Hemocytes were mounted with anti-fading agent (Roti®-Mount Fluorcare, Carl Roth) and observed with a microscope Axioplan2 (Carl Zeiss) equipped for epifluorescence microscopy and differential interference contrast optics. Images were collected with a digital Axiocam (Carl Zeiss).

### Bacterial challenges

Adult LL01 and YR2 aphids were injected with approximately 300 *E. coli* (DH5 a) in insect Schneider medium (Sigma-Aldrich) using a nanoinject II nanoinjector (Drummond Scientific Company) under a dissecting microscope.

To study changes in MIF gene expression of LL01 aphids during secondary symbiont infections, 12-days-old aphids from the lineages YR2-Ri, YR2-Hd or YR2-Ss were pricked in the abdomen to collect hemolymph in a total volume of 200 μL aphid Schneider medium (Sigma-Aldrich). Pelleted secondary symbionts were resuspended in 50 μL of insect Schneider medium and counted under a microscope, using a counting chamber. Approximately 300 facultative secondary symbionts were injected in each adult LL01 aphid using a nanoinject II nanoinjector (Drummond Scientific Company) under a dissecting microscope. Control aphids were injected with sterile Schneider medium. Injected aphids were either frozen at -80°C at one and 4 days post-injection, or allowed to deposit nymphs for 2 days. 12-days-old adults from the first generation (F1) of injected aphids were collected and stored at -80°C until RNA extraction.

### Production of aphids parasitized by *Aphidius ervi*

Three days old aphid nymphs from LL01, YR2, YR2-Ri, YR2-Hd or YR2-Ss were individually exposed to a single *Aphidius ervi* female wasp for 24 hr. Aphids were collected 3 days after parasitoid exposure and stored at -80°C until RNA extraction. For each sample, a set of aphids was maintained on plants under normal conditions to observe mummification and validate the success of parasitoid infection.

### Real-time quantitative reverse transcriptase-PCR (RT-qPCR)

Total RNA was isolated from homogenized tissues (whole bodies or hemocytes) using the RNeasy micro plus kit (Qiagen) and quantified with a nanodrop (Agilent). First-strand cDNA was generated from 500 ng RNA using iScript cDNA synthesis kit, according to standard procedures (Biorad, California, USA). Gene specific primers were designed with Primer3 software [50]. Real-time quantitative PCR was performed on a DNA Engine 2 (MJ Research, Minnesota, USA) with qPCR MasterMix Plus for SYBR green I (Eurogentec, Belgium) using one internal reference transcript (elongation factor 1-α, NCBI RefSeq XM_001951252.2). Running parameters were 95°C for 10 min, followed by 40 cycles of 95°C for 30 s, 60°C for 30 s, 68°C for 30 s. All amplifications were performed in triplicate assays. Signal intensity was measured at the end of each elongation phase and results were analyzed using the Opticon 3.1 software provided by MJ Research. To assess the specificity of the PCR amplification, a melting curve analysis of the amplicon was performed at the end of each reaction and a single peak was always observed. Standard curves were established with four serial dilutions of first-strand cDNAs, ranging from 1/10 to 1/10000. ApMIFs expression levels were normalized to expression of the internal control elongation factor 1-α (EF1) using the comparative CT method (Applied biosystems, USA).

### Statistical analyses

All experiments were repeated three times independently, and each sample corresponds to pools of 10–15 individuals. Differences in relative ApMIFs gene expression levels were tested for statistical significance by one-way ANOVA and the tukey-Kramer test on row data (Software Prism v.5.0, GraphPad). Expression of the target genes are presented either as an expression ratio normalized to EF1 expression or as a relative expression normalized to EF1 expression and to expression in the control sample.

### Availability of supporting data

The data set supporting the phylogenetic analysis is available in the TreeBase repository (http://purl.org/phylo/treebase/phylows/study/TB2:S16212).

## Electronic supplementary material

Additional file 1: Figure S1: Alignment of macrophage migration inhibitory factor (MIFs) sequences. Sequences originate from *Homo sapiens* Hs-MIF (genbank AAA21814.1), *Ancylostoma ceylanicum* Ac-MIF (genbank ABO31935.1) and *Acyrthosiphon pisum* Ap-MIF (NP_001156107.1, XP_001946940.1, NP_001119629.1, NP_001155532.1, XP_001948082.1). The N-terminal catalytic proline (Pro2) and other invariant residues (Lys33, Ile65 and Tyr93 and Val107) that form the MIF active site where substrate molecules interact are indicated in red. Asterisks indicate identical residues; colons and dots indicate residues with high and low levels of similarity, respectively. The 12-mer peptides used for the production of anti-MIF1 antibody are underlined. (DOC 24 KB)
