# Supplementary material for: Diversification of MIF immune regulators in aphids: link with agonistic and antagonistic interactions
Source: BMC Genomics. 2014 Sep 5;15(1):762. doi: 10.1186/1471-2164-15-762 (PMC4169804; doi:10.1186/1471-2164-15-762)
Supplement: Supplementary file 1 — Additional file 1: Figure S1: Alignment of macrophage migration inhibitory factor (MIFs) sequences. Sequences originate from Homo sapiens Hs-MIF (genbank AAA21814.1), Ancylostoma ceylanicum Ac-MIF (genbank ABO31935.1) and Acyrthosiphon pisum Ap-MIF (NP_001156107.1, XP_001946940.1, NP_001119629.1, NP_001155532.1, XP_001948082.1). The N-terminal catalytic proline (Pro2) and other invariant residues (Lys33, Ile65 and Tyr93 and Val107) that form the MIF active site where substrate molecules interact are indicated in red. Asterisks indicate identical residues; colons and dots indicate residues with high and low levels of similarity, respectively. The 12-mer peptides used for the production of anti-MIF1 antibody are underlined. (DOC 24 KB) [file 12864_2014_6457_MOESM1_ESM.doc]

Hs-MIF M**P**MFIVNTNVPRASVPDGFLSELTQQLAQATG**K**PPQYIAVHVVPDQLMAFGGSSEPCALC 60

Ac-MIF M**P**MVRVATNLPDKDVPANFEERLTDLLAESMN**K**PRNRIAIEVLAGQRITHGASRNPVAVI 60

Ap-MIF1 M**P**HFRLETNVSKSKVTPEILKKISAAVAKTLG**K**PESYVVVTIVPDQLMHWDGDDKPCGTA 60

Ap-MIF2 M**P**RLSLDTNLPASKIPEDFLSTCTSLLSKSLG**K**RQSYCVSTVNPGVIMTLGGSNDPCGFI 60

Ap-MIF3 M**P**TLSITTNLPKYKIPSTFLADASKLVSQVLQTPELYIAVRIKAGQQMFWYNNESLCALG 60

Ap-MIF4 MSILRIDTNVSHLDIDDAFLVESTEALAKTLK**K**PKSEILVFVNGNQPILIAGSDEPAIIV 60

Ap-MIF5 -MSYTIDTNLAASQVPDGFLSDTSDFLCALFDRPKKVIMGQLRSGQEFDFSGSTDHCVVM 59

: **:. .: : :. : . :

Hs-MIF SLH-----S**I**GKIGGAQNRSYSKLLCGLLAERLRISPDRV**Y**INYYDMNAAN**V**GWNNSTFA 115

Ac-MIF KVE-----S**I**GALSADDNIRHTQKITQFCQDTLKLPKDKVIITYFDLQPIH**V**GFNGTTVA 115

Ap-MIF1 TLM-----S**I**GSLGVEQNKKHAAVLYPLLKKELGIPDDRL**Y**ITFSDQSSSN**V**GYSGTTFQ 115

Ap-MIF2 QVT-----S**I**GSLGPEENPKHIEVLTDYMHQTLGIPKERLLINLQANIQETTGYLGTTFY 115

Ap-MIF3 NLT-----GTGNFGIDENKHYASIIYDFIEKQLGIPQDKF**Y**LSFVEQKPSNIGVRGTTLE 115

Ap-MIF4 SLL-----SVGGINEIDNKLHSAALFSLITKYLKINENRITIAFSPIEPHAMGHNGKMVV 115

Ap-MIF5 NLTRGSLRPNTTTDEEAIERYVAAISEYLQNALGVPKSRIMIFYYEHDMALIGNNGRTLK 119

: . : : . * : ... : . .

Hs-MIF ------------

Ac-MIF AATM-------- 119

Ap-MIF1 TILG-------- 119

Ap-MIF2 QLFTVEKGKRL- 126

Ap-MIF3 EIIQ-------- 119

Ap-MIF4 Q----------- 116

Ap-MIF5 RIWSEIGTFPSD 131
